# Supplementary material for: The Antioxidant Dendrobium officinale Polysaccharide Modulates Host Metabolism and Gut Microbiota to Alleviate High-Fat Diet-Induced Atherosclerosis in ApoE−/− Mice
Source: Antioxidants (Basel). 2024 May 13;13(5):599. doi: 10.3390/antiox13050599 (PMC11117934; doi:10.3390/antiox13050599)
Supplement: Supplementary file 1 [file antioxidants-13-00599-s001.zip › Supplemental figure.pdf]

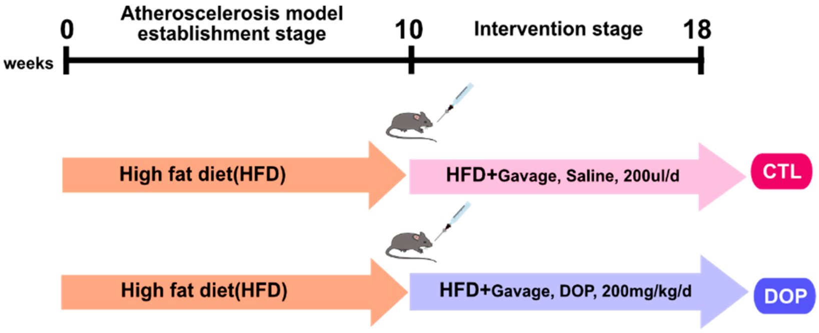

Supplementary Figure S1. Scheme of DOP intervention in HFD-induced atherosclerosis in ApoE$^{-/-}$ mice.

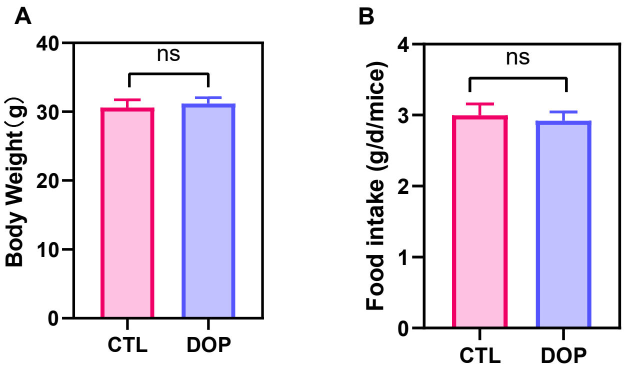

Supplementary Figure S2. Effects of DOP treatments on average body weight (A) and daily food intake (B) in ApoE$^{-/-}$ mice.

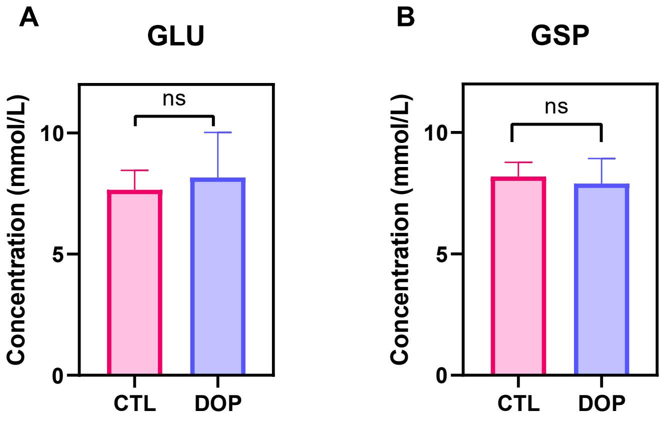

Supplementary Figure S3. Effects of DOP treatments on the levels of glucose (A) and glycosylated serum protein (GSP) (B).
