# Supplementary material for: The Antioxidant Dendrobium officinale Polysaccharide Modulates Host Metabolism and Gut Microbiota to Alleviate High-Fat Diet-Induced Atherosclerosis in ApoE−/− Mice
Source: Antioxidants (Basel). 2024 May 13;13(5):599. doi: 10.3390/antiox13050599 (PMC11117934; doi:10.3390/antiox13050599)
Supplement: Supplementary file 1 [file antioxidants-13-00599-s001.zip › Supplemental table.pdf]

Supplementary Table S1. Target genes and primer sequences

Genes	Accession number	Forward (5′-3′)	Reverse (5′-3′)
Tnf-a	NM_013693	GCCGGACTCATCGTACTCC	TTTGCTACGACGTGGGCTAC
Il1-β	NM_008361	TGCCACCTTTTGACAGTGATG	TGCCACCTTTTGACAGTGATG
Il-6	NM_031168	TGCCACCTTTTGACAGTGATG	CGCACTAGGTTTGCCGAGTA
Arg1	NM_007482	CGCACTAGGTTTGCCGAGTA	CGCACTAGGTTTGCCGAGTA
Mrc1	NM_008625	CGCACTAGGTTTGCCGAGTA	TGTACCGCACCCTCCATCTA
Retnla	NM_020509	GGGATGACTGCTACTGGGTG	TCAACGAGTAAGCACAGGCA
Irf4	NM_013674	GCCAGCCCAGGTTCATAACTA	TGGGGCACAAGCATAAAAGGT
β-Actin	NM_007393	GTGACGTTGACATCCGTAAAGA	GCCGGACTCATCGTACTCC

Supplementary Table S2. Molecular weight and monosaccharide composition of DOP

Items	Mount
Number average molecular weight (Mn)	1080
Weight average molecular weight (Mw)	4538
Z average molecular weight (Mz)	34407
Mw/Mn	4.20476
Mz/Mw	7.58021
Glycosyl composition	
Mannose	1.16
glucose	1
